# 2-(5-Meth­oxy-2-methyl-1*H*-indol-3-yl)-*N*′-[(1*E*,2*E*)-3-phenyl­prop-2-en-1-yl­idene]acetohydrazide

**DOI:** 10.1107/S1600536813023805

**Published:** 2013-08-31

**Authors:** Mehmet Akkurt, Joel T. Mague, Shaaban K. Mohamed, Mustafa R. Albayati, Mahmoud A. A. El-Remaily

**Affiliations:** a Department of Physics, Faculty of Sciences, Erciyes University, 38039 Kayseri, Turkey; b Department of Chemistry, Tulane University, New Orleans, LA 70118, USA; c Chemistry and Environmental Division, Manchester Metropolitan University, Manchester M1 5GD, England; d Chemistry Department, Faculty of Science, Minia University, 61519 El-Minia, Egypt; e Kirkuk University, College of Science, Department of Chemistry, Kirkuk, Iraq; f Department of Organic Chemistry, Faculty of Science, Institute of Biotechnology, Granada University, Granada E-18071, Spain; g Department of Chemistry, Sohag University, 82524 Sohag, Egypt

## Abstract

The title compound, C_21_H_21_N_3_O_2_, adopts a J-shaped conformation which appears to be at least partially directed by a weak intra­molecular C—H⋯N hydrogen bond. In the crystal, mol­ecules are linked by N—H⋯O hydrogen bonds into *R*_2_^2^(8) and *R*_2_^2^(14) cyclic dimers, which form a chain running parallel to the *b* axis.

## Related literature

For general background to side-effect toxicity of non-steroidal anti-inflammatory drugs (NSAIDs), see: Agrawal *et al.* (2010 ▶); Champion *et al.* (1997 ▶); Allan & Fletcher (1990 ▶). For reduction of GI toxicity attributed to NSAIDs, see: Halen *et al.* (2009 ▶); Schoen & Vender (1989 ▶); Mitchell & Warner (1999 ▶). For hydrogen-bond motifs, see: Etter *et al.* (1990 ▶).
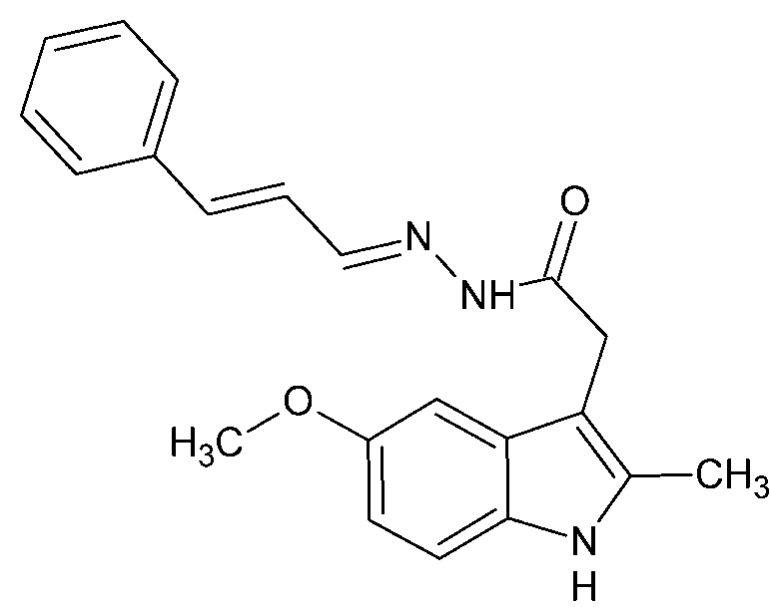


## Experimental

### 

#### Crystal data

C_21_H_21_N_3_O_2_
*M* *_r_* = 347.41
Triclinic, 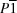

*a* = 8.2786 (9) Å
*b* = 10.1194 (11) Å
*c* = 11.7739 (13) Å
α = 93.001 (2)°
β = 108.993 (2)°
γ = 105.578 (2)°
*V* = 887.76 (17) Å^3^
*Z* = 2
Mo *K*α radiation
μ = 0.09 mm^−1^
*T* = 150 K
0.26 × 0.13 × 0.08 mm

#### Data collection

Bruker SMART APEX CCD diffractometer
Absorption correction: multi-scan (*SADABS*; Bruker, 2013 ▶) *T*_min_ = 0.78, *T*_max_ = 0.99
16303 measured reflections
4574 independent reflections
3675 reflections with *I* > 2σ(*I*)
*R*_int_ = 0.041

#### Refinement

*R*[*F* ^2^ > 2σ(*F* ^2^)] = 0.045
*wR*(*F* ^2^) = 0.126
*S* = 1.08
4574 reflections
245 parameters
H atoms treated by a mixture of independent and constrained refinement
Δρ_max_ = 0.36 e Å^−3^
Δρ_min_ = −0.25 e Å^−3^

### 

Data collection: *APEX2* (Bruker, 2013 ▶); cell refinement: *SAINT* (Bruker, 2013 ▶); data reduction: *SAINT*; program(s) used to solve structure: *SHELXS97* (Sheldrick, 2008 ▶); program(s) used to refine structure: *SHELXL97* (Sheldrick, 2008 ▶); molecular graphics: *ORTEP-3 for Windows* (Farrugia, 2012 ▶) and *PLATON* (Spek, 2009 ▶); software used to prepare material for publication: *WinGX* (Farrugia, 2012 ▶) and *PLATON*.

## Supplementary Material

Crystal structure: contains datablock(s) global, I. DOI: 10.1107/S1600536813023805/qm2100sup1.cif

Structure factors: contains datablock(s) I. DOI: 10.1107/S1600536813023805/qm2100Isup2.hkl

Supplementary material file. DOI: 10.1107/S1600536813023805/qm2100Isup3.cml

Additional supplementary materials:  crystallographic information; 3D view; checkCIF report

## Figures and Tables

**Table 1:** Hydrogen-bond geometry (Å, °)

*D*—H⋯*A*	*D*—H	H⋯*A*	*D*⋯*A*	*D*—H⋯*A*
N1—H1⋯O2^i^	0.891 (18)	2.093 (18)	2.9841 (14)	178.1 (9)
N2—H2⋯O2^ii^	0.903 (16)	2.012 (16)	2.9055 (13)	170.0 (14)
C7—H7⋯N3	0.95	2.54	3.3609 (15)	145

Symmetry codes: (i) 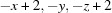; (ii) 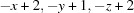.

**Fig. 1.**
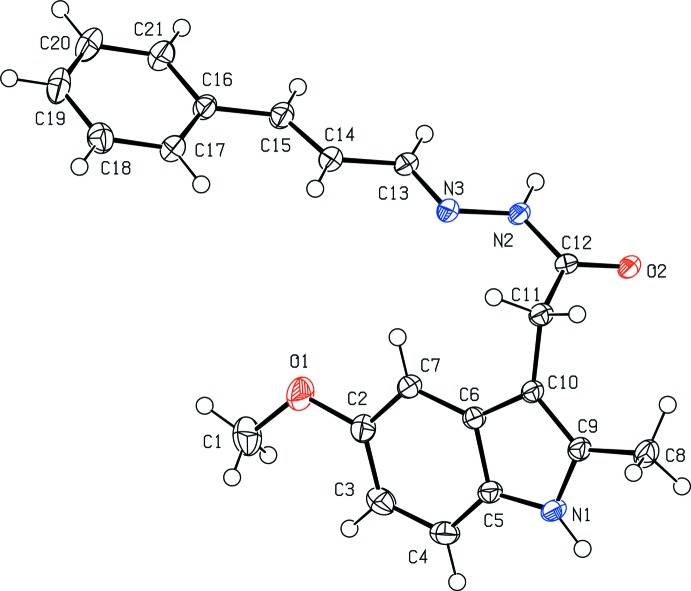
Perspective view of the title molecule with 50% probability displacement ellipsoids.

**Fig. 2.**
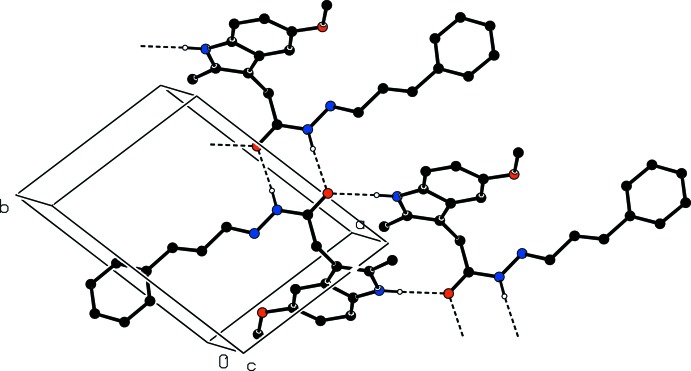
Partial view of the R22(8) and R22(14) cyclic dimers, down the a axis.

**Fig. 3.**
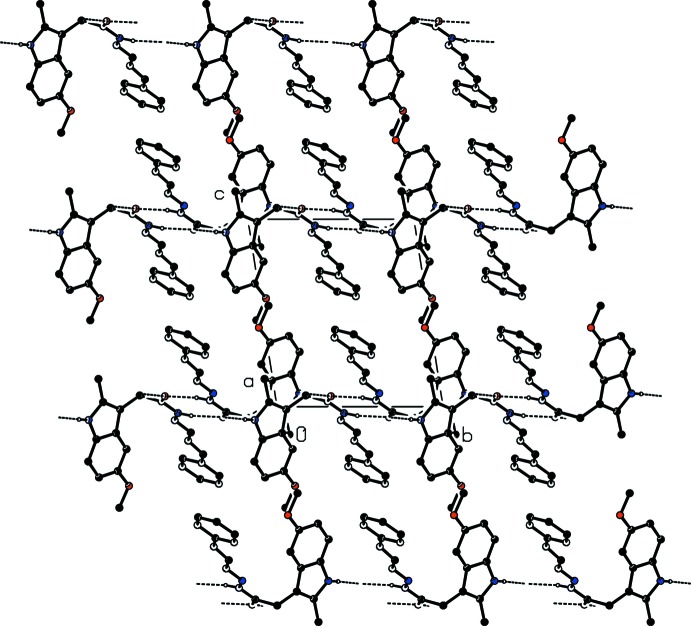
Packing of the title molecule viewed down a with the hydrogen bonds shown by dotted lines.

## supplementary crystallographic information

### 1. Comment

Indomethacin as other common anti-inflammatory drugs (NSAIDs) which are widely employed in the treatment of pain and inflammation has been reported to be associated with a number of undesirable effects, in particular gastrointestinal (GI) toxicity and ulceration (Agrawal *et al.*, 2010; Champion *et al.*, 1997; Allan & Fletcher, 1990) which represent a still unsolved therapeutic problem. Topical irritation by the free carboxylic group of Indomethacin is considered an important factor in establishing superficial stomach erosion (Schoen & Vender, 1989; Mitchell & Warner, 1999). Considerable attention has been focused on the development of bio-reversible derivatives of such pro-drugs to temporarily mask the acidic group as a promising means of reducing or abolishing the GI toxicity due to the local action mechanism (Halen *et al.*, 2009). Based on such facts and continue to our on-going study in functionalization of NSAIDs we herein report the synthesis and crystal structure of the title compound.

The molecular conformation adopted by I in the crystal is "J" shaped (Fig. 1) and appears to be at least partially directed by a weak, intramolecular C7—H7···N3 hydrogen bond. The indole ring system is almostly planar [maximum deviations = -0.046 (1) Å for N1, -0.036 (1) Å for C2 and 0.035 (1) Å for C4] and the dihedral angle between it and the terminal phenyl ring is 79.10 (5)°.

In the crystal structure, the N—H···O hydrogen bonding consists of *R*^2^_2_(8) rings (Etter *et al.*, 1990) with 2 N2—H2···O2 contacts and *R*^2^_2_(14) rings with 2 N1—H1···O2 contacts which form a chain running parallel to the *b* axis (Table 1, Figs. 2 & 3).

### 2. Experimental

A mixture of 233 mg (1 mmol) 2-(5-methoxy-2-methyl-1*H*-indole-3-yl)acetohydrazide and 132 mg (1 mmol) of (2E)-3-phenylprop-2-enal in 50 ml of ethanol containing a few drops of glacial acetic acid was refluxed for 6 hrs. The mixture was cooled to room temperature and the excess solvent was evaporated under *vacuum*. The resulting solid was collected, washed with ethanol and recrystalized from dioxan to give colourless tablets (*M*.p. 410–413 K) suitable for X-ray analysis.

IR (KBr cm^-1^): (C=O amide 1661), (NH 3301), (C=N 1606) (C—H, Ar 3021–3072), (C—H aliphatic 2836–2957). ^1^H-NMR: (DMSO-d_6_) δ at 3.6(s, 3H, –OCH_3_), 2.3(s, 3H, CH_3_), 3.4(s, 2H, –CH_2_), 6.5(d,1*H*, –CH= alkene), 6.6(d,1*H*, –CH= alkene), 8.2(d, 1H, –CH=N), 11.8(s, 1H, –NH amide), the aromatic protons of indole nuclei and benzene ring were appeared in the range of 7.0–7.9. ^13^C-NMR: 161(C=O amide), 149(–CH=N), 55(–OCH_3_), 10(1 C, CH_3_), 136(–C=C alkene), 125(–C=C alkene).

### 3. Refinement

C-bound H atoms were placed in geometrically idealized positions and constrained to ride on their parent atoms with C—H = 0.95–0.99 Å, with *U*_iso_(H) = 1.5 *U*_iso_(C) for methyl H atoms and *U*_iso_(H) = 1.2 *U*_iso_(C) for other H atoms. H atoms bonded to N atoms were located in difference Fourier maps [N1—H1 = 0.891 (18) Å and N2—H2 = 0.903 (16) Å] and refined isotropically.

### Crystal data

**Table:**

C_21_H_21_N_3_O_2_	*Z* = 2
*M**_r_* = 347.41	*F*(000) = 368
Triclinic, *P*1	*D*_x_ = 1.300 Mg m^−^^3^
Hall symbol: -P 1	Mo *K*α radiation, λ = 0.71073 Å
*a* = 8.2786 (9) Å	Cell parameters from 8428 reflections
*b* = 10.1194 (11) Å	θ = 2.6–29.1°
*c* = 11.7739 (13) Å	µ = 0.09 mm^−^^1^
α = 93.001 (2)°	*T* = 150 K
β = 108.993 (2)°	Tablet, clear colourless
γ = 105.578 (2)°	0.26 × 0.13 × 0.08 mm
*V* = 887.76 (17) Å^3^	

### Data collection

**Table:**

Bruker SMART APEX CCD diffractometer	4574 independent reflections
Radiation source: fine-focus sealed tube	3675 reflections with *I* > 2σ(*I*)
Graphite monochromator	*R*_int_ = 0.041
Detector resolution: 8.3660 pixels mm^-1^	θ_max_ = 29.1°, θ_min_ = 1.9°
φ and ω scans	*h* = −11→10
Absorption correction: multi-scan (*SADABS*; Bruker, 2013)	*k* = −13→13
*T*_min_ = 0.78, *T*_max_ = 0.99	*l* = −16→15
16303 measured reflections	

### Refinement

**Table:**

Refinement on *F*^2^	Primary atom site location: structure-invariant direct methods
Least-squares matrix: full	Secondary atom site location: difference Fourier map
*R*[*F*^2^ > 2σ(*F*^2^)] = 0.045	H atoms treated by a mixture of independent and constrained refinement
*wR*(*F*^2^) = 0.126	W = 1/[Σ^2^(*F*O^2^) + (0.0677*P*)^2^ + 0.0983*P*] WHERE *P* = (*F*O^2^ + 2*F*C^2^)/3
*S* = 1.08	(Δ/σ)_max_ = 0.001
4574 reflections	Δρ_max_ = 0.36 e Å^−^^3^
245 parameters	Δρ_min_ = −0.25 e Å^−^^3^
0 restraints	

### Special details

**Table:**

Experimental. The diffraction data were obtained from 3 sets of 400 frames, each of width 0.5° in ω, colllected at φ = 0.00, 90.00 and 180.00° and 2 sets of 800 frames, each of width 0.45° in φ, collected at ω = -30.00 and 210.00°. The scan time was 15 sec/frame.
Geometry. Bond distances, angles *etc*. have been calculated using the rounded fractional coordinates. All su's are estimated from the variances of the (full) variance-covariance matrix. The cell e.s.d.'s are taken into account in the estimation of distances, angles and torsion angles
Refinement. Refinement on *F*^2^ for ALL reflections except those flagged by the user for potential systematic errors. Weighted *R*-factors *wR* and all goodnesses of fit *S* are based on *F*^2^, conventional *R*-factors *R* are based on *F*, with *F* set to zero for negative *F*^2^. The observed criterion of *F*^2^ > σ(*F*^2^) is used only for calculating -*R*-factor-obs *etc*. and is not relevant to the choice of reflections for refinement. *R*-factors based on *F*^2^ are statistically about twice as large as those based on *F*, and *R*-factors based on ALL data will be even larger.

### Fractional atomic coordinates and isotropic or equivalent isotropic displacement parameters (Å^2^)

**Table:**

	*x*	*y*	*z*	*U*_iso_*/*U*_eq_	
O1	0.28193 (13)	0.03509 (10)	0.57833 (8)	0.0383 (3)	
O2	1.01220 (10)	0.33474 (8)	1.05582 (8)	0.0235 (3)	
N1	0.76159 (13)	−0.14643 (9)	0.92901 (9)	0.0233 (3)	
N2	0.76767 (12)	0.39782 (9)	0.96609 (9)	0.0198 (3)	
N3	0.58483 (12)	0.37175 (9)	0.92778 (9)	0.0213 (3)	
C1	0.1780 (2)	−0.04316 (16)	0.46126 (12)	0.0406 (4)	
C2	0.40353 (16)	−0.01850 (12)	0.65687 (11)	0.0261 (3)	
C3	0.44402 (17)	−0.13776 (12)	0.62313 (11)	0.0289 (3)	
C4	0.56565 (16)	−0.18727 (11)	0.70752 (11)	0.0264 (3)	
C5	0.64546 (14)	−0.11740 (10)	0.82602 (11)	0.0210 (3)	
C6	0.61147 (14)	0.00623 (10)	0.85962 (10)	0.0190 (3)	
C7	0.48756 (14)	0.05444 (11)	0.77385 (10)	0.0216 (3)	
C8	0.92662 (16)	−0.04883 (12)	1.14821 (11)	0.0266 (3)	
C9	0.80675 (14)	−0.04222 (11)	1.02468 (11)	0.0212 (3)	
C10	0.71796 (14)	0.05319 (10)	0.98586 (10)	0.0190 (3)	
C11	0.73269 (15)	0.18427 (10)	1.06001 (10)	0.0203 (3)	
C12	0.84755 (14)	0.31009 (10)	1.02798 (10)	0.0184 (3)	
C13	0.52187 (15)	0.45707 (11)	0.86269 (10)	0.0217 (3)	
C14	0.33089 (15)	0.43345 (11)	0.81838 (11)	0.0235 (3)	
C15	0.24928 (15)	0.51098 (12)	0.74611 (11)	0.0245 (3)	
C16	0.05663 (15)	0.49356 (11)	0.70035 (10)	0.0223 (3)	
C17	−0.06817 (16)	0.38488 (12)	0.72362 (11)	0.0257 (3)	
C18	−0.24894 (16)	0.37336 (13)	0.68079 (11)	0.0289 (3)	
C19	−0.30851 (17)	0.47052 (15)	0.61408 (12)	0.0342 (4)	
C20	−0.18776 (18)	0.57806 (15)	0.58911 (12)	0.0350 (4)	
C21	−0.00671 (17)	0.58889 (13)	0.63135 (11)	0.0291 (3)	
H1	0.827 (2)	−0.2041 (17)	0.9322 (15)	0.044 (4)*	
H1A	0.25730	−0.05190	0.41690	0.0610*	
H1B	0.09370	0.00410	0.41640	0.0610*	
H1C	0.11120	−0.13570	0.46960	0.0610*	
H2	0.838 (2)	0.4754 (16)	0.9519 (13)	0.032 (4)*	
H3	0.38760	−0.18480	0.54180	0.0350*	
H4	0.59380	−0.26740	0.68470	0.0320*	
H7	0.46140	0.13600	0.79550	0.0260*	
H8A	0.98240	0.04490	1.19500	0.0400*	
H8B	1.02000	−0.08780	1.14140	0.0400*	
H8C	0.85640	−0.10790	1.18960	0.0400*	
H11A	0.78640	0.17950	1.14750	0.0240*	
H11B	0.61160	0.19340	1.04460	0.0240*	
H13	0.59940	0.53380	0.84430	0.0260*	
H14	0.25880	0.35810	0.84210	0.0280*	
H15	0.32320	0.58470	0.72200	0.0290*	
H17	−0.02820	0.31800	0.76950	0.0310*	
H18	−0.33210	0.29880	0.69720	0.0350*	
H19	−0.43240	0.46330	0.58550	0.0410*	
H20	−0.22870	0.64450	0.54300	0.0420*	
H21	0.07530	0.66240	0.61290	0.0350*	

### Atomic displacement parameters (Å^2^)

**Table:**

	*U*^11^	*U*^22^	*U*^33^	*U*^12^	*U*^13^	*U*^23^
O1	0.0382 (5)	0.0443 (5)	0.0258 (5)	0.0207 (4)	−0.0034 (4)	0.0015 (4)
O2	0.0174 (4)	0.0182 (4)	0.0346 (5)	0.0081 (3)	0.0066 (3)	0.0048 (3)
N1	0.0215 (5)	0.0171 (4)	0.0333 (5)	0.0093 (4)	0.0093 (4)	0.0057 (4)
N2	0.0159 (4)	0.0161 (4)	0.0280 (5)	0.0064 (3)	0.0072 (4)	0.0047 (4)
N3	0.0161 (4)	0.0197 (4)	0.0277 (5)	0.0069 (3)	0.0064 (4)	0.0016 (4)
C1	0.0359 (8)	0.0526 (8)	0.0244 (7)	0.0121 (6)	0.0006 (6)	0.0028 (6)
C2	0.0228 (6)	0.0271 (5)	0.0261 (6)	0.0082 (4)	0.0052 (5)	0.0048 (5)
C3	0.0302 (6)	0.0265 (6)	0.0257 (6)	0.0048 (5)	0.0083 (5)	−0.0029 (5)
C4	0.0277 (6)	0.0190 (5)	0.0338 (7)	0.0063 (4)	0.0138 (5)	0.0002 (4)
C5	0.0181 (5)	0.0161 (5)	0.0297 (6)	0.0054 (4)	0.0095 (4)	0.0040 (4)
C6	0.0162 (5)	0.0162 (4)	0.0252 (6)	0.0045 (4)	0.0084 (4)	0.0034 (4)
C7	0.0201 (5)	0.0191 (5)	0.0264 (6)	0.0074 (4)	0.0078 (5)	0.0041 (4)
C8	0.0227 (6)	0.0250 (5)	0.0329 (6)	0.0098 (4)	0.0076 (5)	0.0126 (5)
C9	0.0174 (5)	0.0180 (5)	0.0285 (6)	0.0057 (4)	0.0075 (4)	0.0071 (4)
C10	0.0165 (5)	0.0163 (4)	0.0244 (5)	0.0054 (4)	0.0068 (4)	0.0048 (4)
C11	0.0210 (5)	0.0183 (5)	0.0227 (5)	0.0075 (4)	0.0076 (4)	0.0044 (4)
C12	0.0190 (5)	0.0159 (4)	0.0206 (5)	0.0075 (4)	0.0059 (4)	0.0004 (4)
C13	0.0205 (5)	0.0191 (5)	0.0265 (6)	0.0085 (4)	0.0076 (5)	0.0024 (4)
C14	0.0199 (5)	0.0220 (5)	0.0278 (6)	0.0080 (4)	0.0065 (5)	0.0021 (4)
C15	0.0204 (5)	0.0242 (5)	0.0301 (6)	0.0088 (4)	0.0084 (5)	0.0059 (4)
C16	0.0212 (5)	0.0248 (5)	0.0223 (5)	0.0115 (4)	0.0059 (4)	0.0019 (4)
C17	0.0239 (6)	0.0260 (5)	0.0281 (6)	0.0116 (4)	0.0071 (5)	0.0043 (5)
C18	0.0230 (6)	0.0335 (6)	0.0282 (6)	0.0074 (5)	0.0082 (5)	0.0003 (5)
C19	0.0224 (6)	0.0487 (8)	0.0317 (7)	0.0172 (5)	0.0049 (5)	0.0051 (6)
C20	0.0320 (7)	0.0429 (7)	0.0341 (7)	0.0226 (6)	0.0066 (6)	0.0131 (6)
C21	0.0272 (6)	0.0319 (6)	0.0307 (6)	0.0136 (5)	0.0089 (5)	0.0095 (5)

### Geometric parameters (Å, º)

**Table:**

O1—C1	1.4195 (17)	C16—C17	1.3991 (17)
O1—C2	1.3793 (16)	C17—C18	1.384 (2)
O2—C12	1.2432 (15)	C18—C19	1.3842 (19)
N1—C5	1.3824 (16)	C19—C20	1.382 (2)
N1—C9	1.3822 (15)	C20—C21	1.388 (2)
N2—N3	1.3758 (15)	C1—H1A	0.9800
N2—C12	1.3512 (15)	C1—H1B	0.9800
N3—C13	1.2866 (15)	C1—H1C	0.9800
N1—H1	0.891 (18)	C3—H3	0.9500
N2—H2	0.903 (16)	C4—H4	0.9500
C2—C7	1.3856 (16)	C7—H7	0.9500
C2—C3	1.4073 (18)	C8—H8A	0.9800
C3—C4	1.3871 (18)	C8—H8B	0.9800
C4—C5	1.3888 (17)	C8—H8C	0.9800
C5—C6	1.4179 (15)	C11—H11A	0.9900
C6—C10	1.4354 (16)	C11—H11B	0.9900
C6—C7	1.3986 (16)	C13—H13	0.9500
C8—C9	1.4869 (17)	C14—H14	0.9500
C9—C10	1.3748 (16)	C15—H15	0.9500
C10—C11	1.5030 (15)	C17—H17	0.9500
C11—C12	1.5169 (15)	C18—H18	0.9500
C13—C14	1.4409 (19)	C19—H19	0.9500
C14—C15	1.3328 (17)	C20—H20	0.9500
C15—C16	1.4645 (19)	C21—H21	0.9500
C16—C21	1.3936 (17)		
			
C1—O1—C2	117.79 (11)	C16—C21—C20	121.01 (13)
C5—N1—C9	109.03 (9)	O1—C1—H1A	109.00
N3—N2—C12	121.25 (9)	O1—C1—H1B	109.00
N2—N3—C13	116.02 (10)	O1—C1—H1C	109.00
C9—N1—H1	121.1 (11)	H1A—C1—H1B	109.00
C5—N1—H1	127.1 (11)	H1A—C1—H1C	109.00
N3—N2—H2	120.7 (11)	H1B—C1—H1C	109.00
C12—N2—H2	118.1 (11)	C2—C3—H3	120.00
O1—C2—C3	123.67 (11)	C4—C3—H3	120.00
O1—C2—C7	115.35 (11)	C3—C4—H4	121.00
C3—C2—C7	120.98 (12)	C5—C4—H4	121.00
C2—C3—C4	120.58 (11)	C2—C7—H7	121.00
C3—C4—C5	118.66 (11)	C6—C7—H7	120.00
C4—C5—C6	121.15 (11)	C9—C8—H8A	109.00
N1—C5—C6	107.51 (10)	C9—C8—H8B	109.00
N1—C5—C4	131.34 (10)	C9—C8—H8C	109.00
C5—C6—C10	106.84 (10)	H8A—C8—H8B	109.00
C7—C6—C10	133.62 (10)	H8A—C8—H8C	109.00
C5—C6—C7	119.50 (10)	H8B—C8—H8C	109.00
C2—C7—C6	119.01 (11)	C10—C11—H11A	109.00
N1—C9—C8	120.64 (10)	C10—C11—H11B	109.00
N1—C9—C10	109.46 (10)	C12—C11—H11A	109.00
C8—C9—C10	129.81 (11)	C12—C11—H11B	109.00
C6—C10—C11	126.11 (10)	H11A—C11—H11B	108.00
C9—C10—C11	126.77 (10)	N3—C13—H13	121.00
C6—C10—C9	107.10 (10)	C14—C13—H13	121.00
C10—C11—C12	110.76 (10)	C13—C14—H14	118.00
N2—C12—C11	118.76 (11)	C15—C14—H14	118.00
O2—C12—N2	118.88 (10)	C14—C15—H15	117.00
O2—C12—C11	122.36 (10)	C16—C15—H15	117.00
N3—C13—C14	118.40 (11)	C16—C17—H17	119.00
C13—C14—C15	123.95 (11)	C18—C17—H17	120.00
C14—C15—C16	126.31 (11)	C17—C18—H18	120.00
C15—C16—C17	122.69 (11)	C19—C18—H18	120.00
C15—C16—C21	119.29 (11)	C18—C19—H19	120.00
C17—C16—C21	118.02 (12)	C20—C19—H19	120.00
C16—C17—C18	121.03 (11)	C19—C20—H20	120.00
C17—C18—C19	119.96 (12)	C21—C20—H20	120.00
C18—C19—C20	119.99 (14)	C16—C21—H21	119.00
C19—C20—C21	119.99 (13)	C20—C21—H21	120.00
			
C1—O1—C2—C3	7.23 (19)	C5—C6—C10—C11	176.91 (11)
C1—O1—C2—C7	−173.47 (12)	C7—C6—C10—C9	176.05 (13)
C9—N1—C5—C4	178.38 (13)	C7—C6—C10—C11	−5.5 (2)
C9—N1—C5—C6	−2.32 (13)	N1—C9—C10—C6	0.16 (14)
C5—N1—C9—C8	178.24 (11)	N1—C9—C10—C11	−178.30 (11)
C5—N1—C9—C10	1.36 (14)	C8—C9—C10—C6	−176.35 (12)
C12—N2—N3—C13	−175.51 (10)	C8—C9—C10—C11	5.2 (2)
N3—N2—C12—O2	176.74 (10)	C6—C10—C11—C12	−74.62 (15)
N3—N2—C12—C11	−2.72 (16)	C9—C10—C11—C12	103.55 (13)
N2—N3—C13—C14	179.00 (10)	C10—C11—C12—O2	−70.23 (14)
O1—C2—C3—C4	−178.89 (12)	C10—C11—C12—N2	109.21 (12)
C7—C2—C3—C4	1.8 (2)	N3—C13—C14—C15	−177.08 (12)
O1—C2—C7—C6	179.33 (11)	C13—C14—C15—C16	−178.72 (11)
C3—C2—C7—C6	−1.35 (19)	C14—C15—C16—C17	−3.5 (2)
C2—C3—C4—C5	0.5 (2)	C14—C15—C16—C21	176.09 (12)
C3—C4—C5—N1	175.97 (13)	C15—C16—C17—C18	178.73 (11)
C3—C4—C5—C6	−3.25 (19)	C21—C16—C17—C18	−0.85 (18)
N1—C5—C6—C7	−175.64 (11)	C15—C16—C21—C20	−178.26 (12)
N1—C5—C6—C10	2.37 (13)	C17—C16—C21—C20	1.33 (18)
C4—C5—C6—C7	3.75 (18)	C16—C17—C18—C19	−0.17 (19)
C4—C5—C6—C10	−178.25 (11)	C17—C18—C19—C20	0.7 (2)
C5—C6—C7—C2	−1.38 (17)	C18—C19—C20—C21	−0.3 (2)
C10—C6—C7—C2	−178.75 (13)	C19—C20—C21—C16	−0.8 (2)
C5—C6—C10—C9	−1.55 (13)		

### Hydrogen-bond geometry (Å, º)

**Table:**

*D*—H···*A*	*D*—H	H···*A*	*D*···*A*	*D*—H···*A*
N1—H1···O2^i^	0.891 (18)	2.093 (18)	2.9841 (14)	178.1 (9)
N2—H2···O2^ii^	0.903 (16)	2.012 (16)	2.9055 (13)	170.0 (14)
C7—H7···N3	0.95	2.54	3.3609 (15)	145
C11—H11*B*···N3	0.99	2.34	2.7996 (15)	107

Symmetry codes: (i) −*x*+2, −*y*, −*z*+2; (ii) −*x*+2, −*y*+1, −*z*+2.
